# Theranostics Nuclear Medicine in Prostate Cancer

**DOI:** 10.3390/ph17111483

**Published:** 2024-11-05

**Authors:** Helena Lima, Marina Etchebehere, Mateos Bogoni, Caroline Torricelli, Ellen Nogueira-Lima, Victor M. Deflon, Mariana Lima, Elba Etchebehere

**Affiliations:** 1 Faculdade de Medicina, Pontifícia Universidade Católica de Campinas (PUCC), Campinas 13087-571, Brazil; he.heleninha@gmail.com; 2 Faculdade Israelita de Ciências da Saúde Albert Einstein, Hospital Israelita Albert Einstein, São Paulo 05652-000, Brazil; etchebeheremarina@gmail.com; 3 Hospital Erasto Gaertner, Curitiba 81520-060, Brazil; bogonimateos@gmail.com; 4 Diagnóstico Avançado por Imagem (DAPI), Curitiba 80430-210, Brazil; 5 Division of Nuclear Medicine, University of Campinas (UNICAMP), Campinas 13083-888, Brazil; caroltorricelli@gmail.com (C.T.); ellen.nogueira.lima@gmail.com (E.N.-L.); marlima1@unicamp.br (M.L.); 6 São Carlos Institute of Chemistry, University of São Paulo (USP), São Carlos 13566-590, Brazil; deflon@iqsc.usp.br; 7 Medicina Nuclear de Campinas (Grupo MND), Campinas 13020-432, Brazil

**Keywords:** PET/CT, PSMA, ^18^F-FDG, 18F-fluoride, Ra-223, ^177^Lu-PSMA, nuclear medicine

## Abstract

Theranostic Nuclear Medicine is based on the idea of combining the same molecule (or drug) with different radioisotopes for both diagnosis and treatment, a concept that emerged in the early 1940s with the use of radioactive iodine for thyroid diseases. Theranostic Nuclear Medicine has since expanded to diseases of higher incidence, such as prostate cancer, with several imaging methods used to assess the extent of the disease and the corresponding radiopharmaceuticals used for treatment. For example, by detecting osteoblastic metastases by bone scintigraphy, corresponding radiopharmaceuticals with therapeutic properties can be administered to eliminate or reduce pain associated with metastases and/or determine overall survival gain. The purpose of this review is to discuss the role of Theranostic Nuclear Medicine in prostate cancer, addressing the main diagnostic imaging studies with their corresponding treatments in the Theranostic model.

## 1. Introduction

Theranostic Nuclear Medicine is based on the idea of combining the same molecule (drug) with different radioisotopes, both for diagnosis and treatment. The term theranostic emerged in the late 20th century and its application is on the rise. The term comes from the Greek words *therapeia* (act of healing, performing medical treatment), and *gnosis* (knowledge) [1].

The concept of Theranostic Nuclear Medicine is not new in nuclear medicine, as it is a part of the specialty’s history. In 1947, Seidlin et al. published the first treatment of thyroid cancer with high doses of radioactive iodine, an isotope also used for the diagnosis of thyroid cancer when lower doses are applied [2].

Therefore, the main purpose of Theranostic Nuclear Medicine is to use a radiopharmaceutical with diagnostic properties (for example, containing a low-energy gamma photon-emitting radioisotope) to determine the extent of the disease, and subsequently replace that radioisotope with one with therapeutic properties (high energy photon-emitting radioisotopes) that will be linked to the same drug for treatment.

In a broader definition, we might include the use of different pharmaceuticals and radioisotopes in the diagnostic and therapeutic stages in the Theranostic model, while still under the umbrella of Nuclear Medicine techniques, as we will explore in the management of osteoblastic metastases.

**Figure 1:**
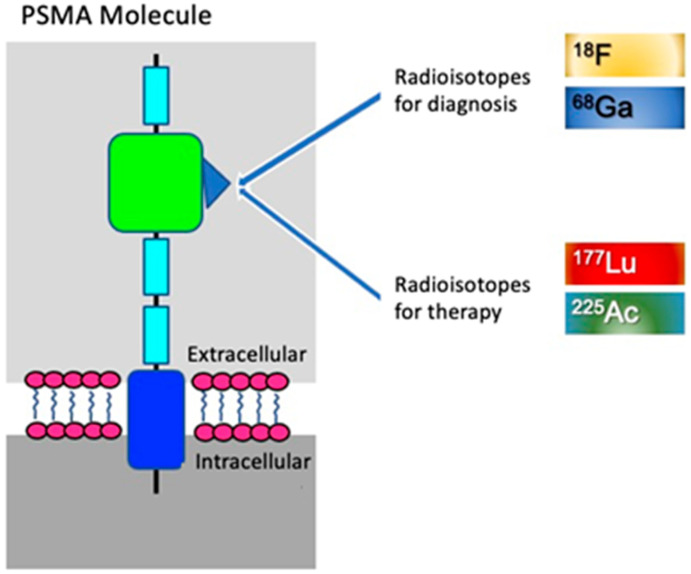
The concept of Theranostics in nuclear medicine, highlighting the structure of prostate-specific membrane antigen (PSMA). The large extracellular catalytic domain of PSMA (green) features binding sites for radioisotopes used in prostate cancer diagnosis, such as fluoride-18 (¹⁸F) and gallium-68 (⁶⁸Ga), and for treatment such as lutetium-177 (¹⁷⁷Lu) and actinium-225 (²²⁵Ac). The extracellular domains of PSMA are represented in light blue, the cell membrane lipid bilayer in pink, and the transmembrane domain with its intracellular antibody-binding site, in dark blue. Design adapted from Maurer T et al. (2016) [4].

A molecule, such as the prostate-specific membrane antigen (PSMA), can be labeled with radioisotopes to be used as a radiopharmaceutical for diagnostic or therapeutic purposes. For example, when the PSMA molecule is labeled with positron-emitting radioisotopes such as fluoride-18 (^18^F-PSMA) or gallium-68 (^68^Ga-PSMA), the molecule will have diagnostic properties and images can be generated on PET/CT equipment. The same PSMA molecule may be subsequently labeled with radioisotopes such as lutetium-177 (^177^Lu, beta emitter) or actinium-225 (^225^Ac, alpha emitter) and will thus present therapeutic properties [3], as exemplified in Figure 1.

Diverse radiopharmaceuticals, each with varying diagnostic sensitivity and specificity, are employed in the detection and staging of prostate cancer, as well as in assessing treatment effectiveness and identifying recurrence sites [5].

Numerous PSMA ligands for labeling with ^68^Ga and ^18^F have been developed, and specialists are actively investigating imaging using ^68^Ga-PSMA-11 and ^18^F-PSMA-1007 [6]. Both PSMA radioligands have similar performance characteristics in positron emission tomography (PET) scans for prostate cancer since they provide high sensitivity and specificity for identifying metastatic lesions [7].

^68^Ga-PSMA positron emission tomography/computed tomography (PET/CT) is the current benchmark for PSMA imaging in prostate cancer for accurately detecting and staging the disease [6]; it detects small metastases or recurrences due to its strong binding affinity, quick intracellular accumulation, and fast clearance from non-target tissues [8]. Alternatively, the physical characteristics of ^18^F radionuclides are superior compared to ^68^Ga-labeled tracers, including a longer half-life, a shorter positron range, and improved image resolution [9,10].

In situations where ^68^Ga-PSMA PET/CT is unavailable due to resource-limited settings, ^99m^Tc-PSMA single-photon emission computed tomography (SPECT/CT) and ^99m^Tc methyl diphosphonate (^99m^Tc-MDP) bone scans are frequently used for staging [6].

PSMA is highly overexpressed in most prostate cancer cells. It also serves as a viable target for radioligand therapies [11]. When combined with ^177^Lu or ^225^Ac, it functions as a theranostic radiotracer, identifying and selectively eliminating prostate cancer cells. This strategy, utilizing alpha or beta-emitting agents, has shown promising results and has become part of personalized medicine, aiding in treatment decision-making and therapy monitoring [12].

Theranostic agents for prostate cancer treatment were initially developed to focus on bone-specific conditions [13]. Radium-223 (^223^Ra) targeted alpha particle therapy selectively targets bone metastases. The US Food and Drug Administration (FDA) has approved it for the treatment of patients with metastatic castration-resistant prostate cancer (mCRPC) [14], and a survival benefit has been demonstrated [13].

Also, samarium-153-EDTMP (^153^Sm-EDTMP), one of the most frequently used bone-targeting beta-emitter radiopharmaceuticals, was primarily employed for palliative treatment in painful bone metastases. It binds to a ligand that attaches to the bone matrix and concentrates in areas of increased bone remodeling. This allows radiation to be delivered directly to osteoblastic metastatic sites [15].

Theranostic Nuclear Medicine has shown remarkable growth in recent years, and the concept has been expanded to diseases such as prostate cancer, one of the illnesses that most affects the male population.

## 2. Principles of Nuclear Medicine

Nuclear medicine uses radioactive materials for diagnosis and therapy through safe and noninvasive methods. It requires two essential components for operation: radiopharmaceuticals and imaging equipment that detects radiation.

Radiopharmaceuticals are composed of radioactive elements (radioisotopes) linked to a specific molecule (pharmaceutical). They are usually injected intravenously, but they can be administered by several means, depending on the clinical indication and the disease to be investigated; for example, they can be inhaled, or administered intrathecally, peritoneally, and orally. After the radiopharmaceutical is concentrated in the specific cell, organ, or organs, images are acquired using nuclear medicine equipment.

Two types of equipment are used to evaluate prostate cancer using nuclear medicine, as described below.

### 2.1. Conventional Scintigraphy and SPECT/CT

**Figure 2:**
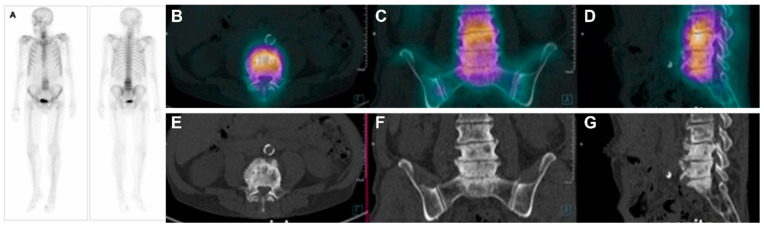
Example of conventional bone scintigraphy and SPECT/CT using the radiopharmaceutical ^99m^Tc-MDP for primary staging of a prostate cancer patient (Gleason score 8). (**A**). Whole-body conventional images demonstrate abnormal uptake in the 3rd, 4th, and 5th lumbar spines. The abnormal uptake was equivocal for metastases. Therefore, the patient underwent SPECT/CT images. The fused axial (**B**), coronal (**C**), and sagittal (**D**) images demonstrate diffuse uptake in the lumbar spine with increased activity in areas consistent with degenerative processes, clearly seen in the CT images in the axial (**E**), coronal (**F**), and sagittal (**G**) planes. Therefore, the patient had degenerative processes and no osteoblastic bone metastases in this study.

Scintillation cameras (or gamma cameras) detect gamma radiation emitted by radiopharmaceuticals. With this nuclear medicine diagnostic imaging equipment, it is possible to evaluate biochemical processes by scanning the entire body, such as conventional bone scintigraphy for detecting osteoblastic metastases in prostate cancer. Conventional bone scintigraphy (or bone scan) has evolved to single photon emission computed tomography (SPECT) images. While in conventional scintigraphy, the obtained images are ‘planar,’ resembling a radiography incidence, SPECT images are volumetric, which means they can be reconstructed tridimensionally, thus providing axial, coronal, and sagittal sequential slices, similar to computed tomography (CT) images. When coupled with computed tomography, hybrid SPECT/CT images are acquired. In SPECT/CT equipment, it is possible to evaluate both the biochemical processes (SPECT) and anatomy (CT) simultaneously through the fusion of SPECT images with CT images (Figure 2). The radiopharmaceutical methylene-bisphosphonate labeled with metastable technetium-99 (^99m^Tc-MDP) is most used in conventional bone scintigraphy and SPECT/CT, and it will be later discussed in further detail.

### 2.2. PET/CT Scan

**Figure 3:**
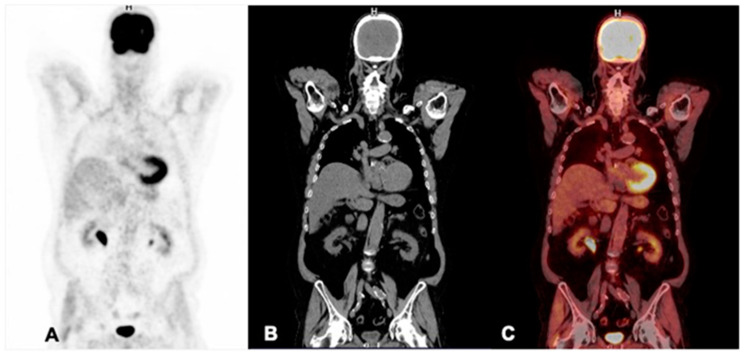
Example of PET/CT images using the radiopharmaceutical ^18^F-FDG of a prostate cancer patient. The PET/CT images allow the simultaneous evaluation of biochemical and biological processes of the whole body (through the PET component) and anatomy (through the CT component). The metabolic images of PET are fused with the anatomic images of the CT scan. The coronal images of the (**A**) PET, (**B**) CT, and fused (**C**) PET/CT images demonstrate normal uptake and no metastases in this study.

Positron emission tomography coupled with computed tomography (PET/CT) is a diagnostic imaging equipment used in nuclear medicine that explores positron-emitting radiopharmaceuticals. These positrons collide with electrons in the patient’s body, in a process called “annihilation”, which results in the simultaneous emission of two gamma photons in opposite directions. The detection of the gamma radiation from these events is the basis of PET images. Similar to SPECT/CT, PET/CT also allows the simultaneous evaluation of biochemical and biological processes in the entire body (via the PET component) and of the anatomy (via the CT component). Molecular PET images are merged with anatomical CT images (Figure 3). The main positron-emitter radiopharmaceuticals used in prostate cancer in Brazil are:

1. Fluorine-18 labeled prostate membrane specific antigen (^18^F-PSMA)
2. Gallium-68 labeled prostate membrane specific antigen (^68^Ga-PSMA)
3. Sodium Fluoride-^18^F (^18^F-fluoride)
4. Fluorine-18 labeled 2-Fluoro-D-deoxy-glucose (^18^F-FDG)

Some of the above-mentioned radiotracers can be applied in the Theranostic model. Below we will discuss Theranostic applications available in the country.

## 3. Theranostics for Osteoblastic Metastases

Osteoblastic metastases from prostate cancer can be diagnosed through bone scintigraphy with or without SPECT/CT, using the radiopharmaceutical ^99m^Tc-MDP, or through PET/CT with the radiopharmaceutical ^18^F-fluoride [16].

In the Theranostic concept, for such tracers, there are corresponding radiopharmaceuticals with therapeutic properties to eliminate or reduce the pain of osteoblastic metastases, such as radium-223 (^223^Ra-Xofigo^®^) and samarium-153 coupled to tetra-ethylene-diamine-methylene-phosphonate (^153^Sm-EDTMP) [17].

Diagnostic tests and corresponding treatments are discussed below.

### 3.1. Diagnosis with Bone Scintigraphy (SPECT/CT) with ^99m^Tc-MDP

The detection of osteoblastic metastases of prostate cancer can be obtained with ^99m^Tc-MDP. Approximately 3 h after venous injection of ^99m^Tc-MDP, this radiotracer concentrates in osteoblastic metastases through adsorption to the hypomineralized bone matrix, exchanging with the phosphate present in hydroxyapatite.

**Figure 4:**
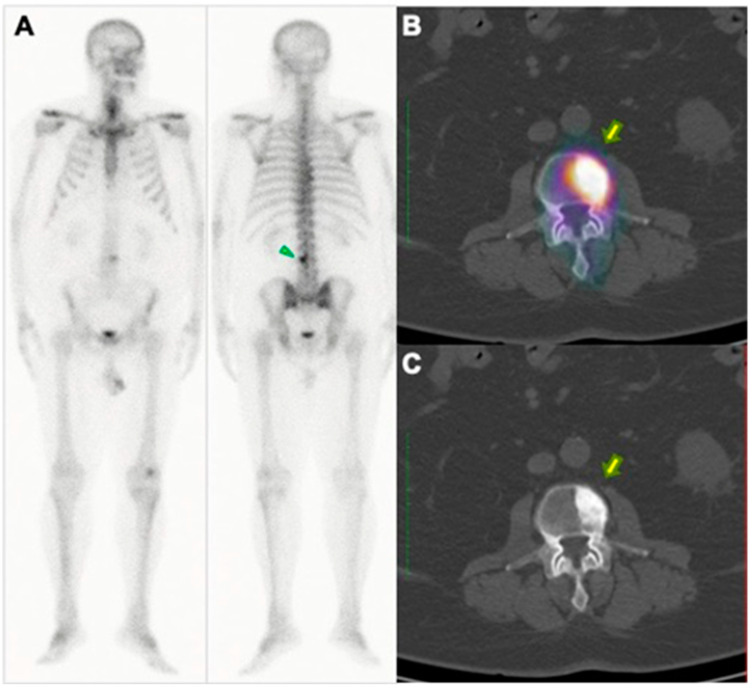
Example of conventional bone scintigraphy and SPECT/CT using the radiopharmaceutical ^99m^Tc-MDP of a 62-year-old prostate cancer patient (Gleason, 3 + 4; PSA equals 11.3 ng/dL) undergoing the evaluation for primary staging. (**A**) Whole-body conventional images demonstrate a focal area of abnormal uptake (arrow) in the 3rd lumbar spine. The abnormal uptake was equivocal for metastases. Therefore, the patient underwent SPECT/CT images. The axial images in the fused SPECT/CT (**B**) and corresponding CT (**C**) demonstrate increased activity in areas consistent with osteoblastic bone metastasis.

The higher the osteoblastic activity (present in metastases), the greater the bone remodeling and, therefore, the greater the concentration of ^99m^Tc-MDP within the metastases (Figure 4). For this reason, the technique is not usually considered adequate for the evaluation of osteolytic lesions, since these are characterized by bone destruction/resorption and, hence, present low bone turnover [18].

### 3.2. Diagnosis with PET/CT with ^18^F-Fluoride

Osteoblastic metastases may be identified with the radiopharmaceutical ^18^F-fluoride. Forty-five minutes after the intravenous injection, it will concentrate on osteoblastic metastases through exchange with the hydroxyl group present in the molecular composition of hydroxyapatite. The greater the osteoblastic activity present in the metastases, the greater the accumulation of ^18^F-fluoride [19].

**Figure 5:**
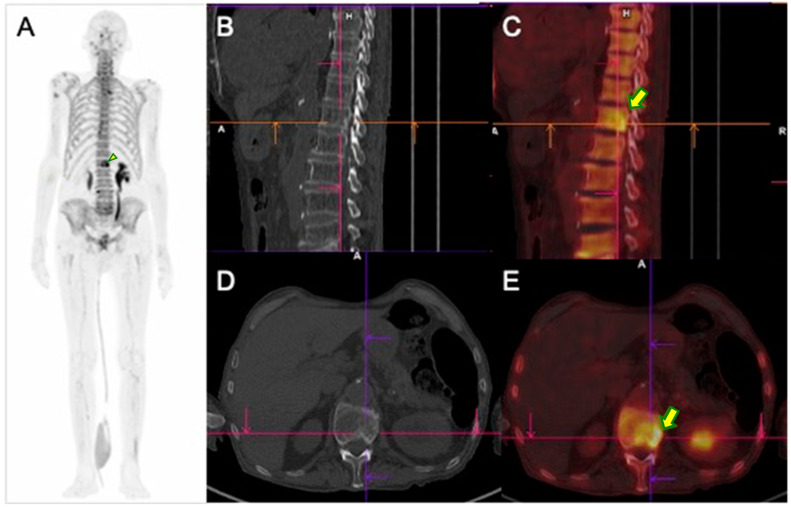
Example of PET/CT using the radiopharmaceutical ^18^F-fluoride of an 85-year-old prostate cancer patient. The patient underwent the study for restaging purposes (Gleason, 4 +3; post-prostatectomy and radiotherapy) due to biochemical recurrence (PSA = 323 ng/dL). (**A**). Whole-body MIP (Maximum Intensity Projection) image demonstrates a focal area of abnormal uptake in the 1st lumbar vertebrae (arrowhead). The sagittal in the CT and fused images in the sagittal (**B**,**C**) and axial (**D**,**E**) images demonstrate increased activity in areas consistent with osteoblastic bone metastasis (yellow arrows).

PET/CT is used to identify ^18^F-fluoride concentration in the skeleton (Figure 5). Compared to conventional bone scintigraphy, PET/CT with ^18^F-fluoride is superior for evaluating the tumor burden of osteoblastic metastases [20,21,22].

When available, ^18^F-fluoride PET/CT should be the imaging study of choice to identify osteoblastic metastases, since it is more accurate and sensitive than conventional bone scintigraphy and has several advantages. For instance, it allows absolute quantification of the tumor metabolic burden, improves patient management as it is an imaging biomarker with prognostic power, reduces waiting time for imaging when compared to conventional bone scintigraphy, and may help guide therapeutic monitoring [16,20,21,22,23,24,25]. Another potential advantage is the simultaneous acquisition of computed tomography (CT) images, allowing the detection of metastases in soft tissues (lymph nodes and viscera) and thereby increasing specificity, since not all SPECT machines are hybrid (i.e., SPECT/CT), as the vast majority of PET equipment are in fact PET/CT.

^18^F-fluoride has similar uptake properties to the therapeutic radiotracer ^223^Ra, making it possible to accurately assess therapeutic efficacy within the Theranostic concept [26].

### 3.3. Treatment with Radium-223

^223^Ra treatment is indicated for selected patients with osteoblastic metastases, which may be identified by conventional bone scan or PET/CT bone imaging [27].

^223^Ra is a calcium analog and, in a similar mechanism to diagnostic tracers such as ^99m^Tc-MDP and ^18^F-fluoride, ^223^Ra adsorbs to the hypomineralized matrix and replaces calcium ions present in hydroxyapatite. In metastatic osteoblastic lesions, ^223^Ra induces calcium replacement through its interaction with osteoblasts, responsible for bone formation at the lesion sites [28].

**Figure 6:**
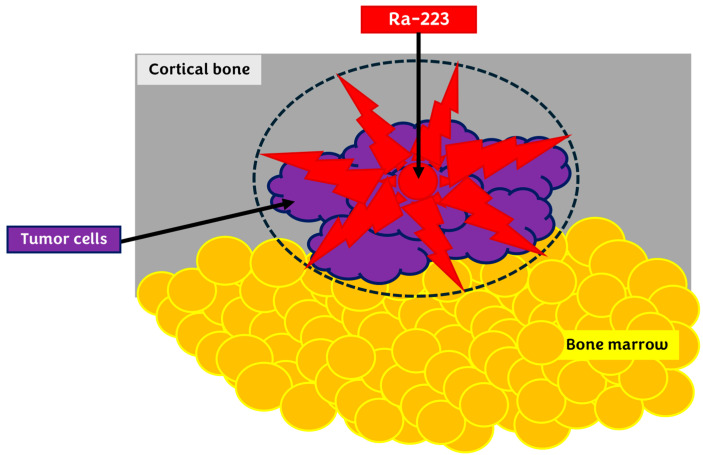
Emission of alpha particles by Ra-223. These high-energy particles follow a very short distance after emission, destroying tumor cells without irradiating surrounding bone marrow and causing less toxicity. Cellular destruction causes irreversible events in the tumor cell by double-stranded DNA breakage, leading to apoptosis and necrosis.

^223^Ra relies on the emission of alpha particles, which are high-energy particles equivalent to a helium atom nucleus (i.e., two protons and two neutrons). These particles travel a very short distance after their emission from the ^223^Ra nucleus. This provides great efficiency for destroying tumor cells without irradiating and destroying surrounding healthy cells, causing less toxicity. Such irradiation occurs even at lower radioactive concentrations due to its high linear energy transfer (LET), a characteristic phenomenon of high-energy radioisotopes, which, in theory, could also promote abscopal effects (therapeutic effects distant from the irradiated targets) (Figure 6). The alpha particles cause irreversible cell death by inducing double-strand DNA break, leading to apoptosis and necrosis [14,26,27].

Currently, ^223^Ra is an established radioisotope in prostate cancer and is widely used in clinical practice. It was approved by the Food and Drug Administration in the United States in 2013 and by the European Medicines Agency in 2018 [15].

Regarded as a suitable option for the management of castration-resistant patients with symptomatic osteoblastic metastases, it is important to highlight its selection criteria: it is best suited for patients with bone-only or bone-predominant disease, with the main guidelines on the subject recommending against its routine administration for patients with visceral metastases or with malignant lymph nodes larger than 3 cm.

Even though symptomatic relief may be expected, the main goal of the treatment is to improve overall survival. To this end, prospective trials and real-world data tend to suggest the necessity of early administration among the lines of treatment in castration-resistance patients. Patients who are given ^223^Ra as the first line of treatment in this setting present better outcomes than those for whom it is given as a second or third line treatment. This seems to correlate with the completion of the full treatment regimen: six cycles of ^223^Ra administered intravenously at doses of 50 kBq/kg (1.35 µCi/kg), once every four weeks. From a radiation safety perspective, the small range of action of alpha-particles favors administration in an outpatient setting, with the whole process taking approximately one hour. After discharge, the patient may resume their normal daily activities.

A multidisciplinary approach to therapy is important, since several details about patient management might interfere with its success. Special attention must be given to bone health, since adverse events such as fractures are not uncommon. Administration of bone-supportive agents, like denosumab or bisphosphonates, is encouraged and is associated with better outcomes.

Monitoring response to the treatment can be challenging. Even though, for most prostate cancer treatments, PSA monitoring is somewhat of a hallmark, ^223^Ra has only a modest effect on serum PSA levels. In fact, an increase in PSA levels can be observed in some cases and does not imply that the treatment is not effective, nor that it should be suspended. Other biomarkers might be more useful, such as ALP (alkaline phosphatase) and LDH (lactate dehydrogenase). From an imaging standpoint, bone scans can be the method of choice for patient selection, but their value for treatment response is limited, which is also true for CT. PET/CT PSMA and whole-body diffusion–weighted magnetic resonance imaging (DWI) hold the promise for more accurate response monitoring, but more robust data are still needed.

Since ^223^Ra is reserved for the later stages of the disease, it is frequently weighed against or in combination with other treatment options, such as external beam radiation therapy (EBRT), new generation anti-androgenic therapies (abiraterone, apalutamide, enzalutamide), chemotherapy, or even PSMA ligands such as ^177^Lu-PSMA. Unfortunately, the use of ^223^Ra combined with these treatment strategies lacks data from prospective trials. Overall, a combination with ERBT is considered feasible and safe. Combination/sequencing with new generation anti-androgenic therapies still awaits prospective trial results but this approach has already highlighted the importance of bone-supportive agents, and since there is limited benefit to sequential treatment with different types of hormonal agents (such as following abiraterone with enzalutamide, for example), ^223^Ra might be a feasible interim option. Combination or sequencing with chemotherapy (notably docetaxel) or PSMA ligands is also not yet established, primarily because of safety concerns regarding the potential for myelosuppression.

A few markers related to poor ^223^Ra prognosis might be considered for the selection of alternative treatments, among which are PSA doubling time <6 months, response to androgen deprivation therapy <12 months, elevated LDH, and extensive lymph node metastases. In these cases, chemotherapy probably represents a more suitable strategy.

### 3.4. Treatment with ^153^Sm-EDTMP

^153^Sm-EDTMP has been used in Brazil since 1995 to treat bone pain caused by osteoblastic metastases from prostate cancer. Its mechanism of action for pain relief is not completely understood. The hypothesis is that there is a reduction of intramedullary pressure that begins a few days after administration of the radiopharmaceutical [15,28].

^153^Sm-EDTMP, like ^223^Ra, targets osteoblastic metastases. However, its accumulation does not occur through exchange with calcium ions, but through its adsorption to the bone matrix and exchange with the phosphate present in hydroxyapatite. Therefore, pain treatment with ^153^Sm-EDTMP is indicated for selected patients who present with bone scintigraphy or bone PET/CT demonstrating osteoblastic metastases, as per the Theranostic concept.

Unlike ^223^Ra, which emits alpha particles, ^153^Sm is a beta particle emitter. Despite releasing high-energy particles, the energy from these particles is 100 to 1000 times lower than that of alpha particles. Furthermore, unlike ^223^Ra, which does not travel far, ^153^Sm has a larger affected tissue range, which provides adequate effectiveness. However, it irradiates neighboring healthy cells at a greater rate and destroys tumor cells at a lesser rate, causing greater toxicity.

Treatment with ^153^Sm-EDTMP is effective as it significantly reduces the use of opioids. Patients with metastatic prostate cancer can remain opioid-free for months with improved quality of life. The consequence is the reduction of morbidity and expenses related to opioids, which brings benefits in terms of public health [29,30].

## 4. Linear Energy Transfer (LET)

LET is the amount of energy deposited by an ionizing particle along its path per unit length, which can affect the inhibition of cell division in microorganisms [31]. The importance of LET properties in cancer patients’ treatments (such as ^223^Ra, ^177^Lu, and ^225^Ac) determines the effectiveness of targeting and killing cancer cells. Additionally, knowing the LET properties of radiopharmaceuticals can predict adverse reactions in the surrounding healthy cancer tissues [32].

Beta-emitting radiopharmaceuticals are efficient in tumor ablation; however, due to their physical properties, they have limitations when it comes to treating some metastatic cancers. Beta particles can travel up to a centimeter from the decay site, which can cause nonspecific irradiation of adjacent healthy tissues. Low LET reduces cytotoxicity; however, it requires a higher radiation dose to have a favorable therapeutic effect. Considering this, beta emitter drugs are less effective in targeting individual cells, small tumor burdens, and micrometastases [33].

On the other hand, alpha particles have significantly higher LET than beta particles (≈50–200 keV/µm vs. ≈0.2–0.4 keV/µm) [34]. Considering this, alpha elements offer a favorable alternative to beta particles for tumor destruction due to their shorter travel distances in biological tissue and higher LET. Alpha particles have a range of 40 to 100 µm, confining them to just a few cell diameters and thereby leading to greater specificity for tumor cells. Their high LET results in more ionization events and lethal DNA double-strand breaks. One single alpha particle crossing the cell nucleus can effectively sterilize the tumor; this is in opposition to the thousands of decay events needed when treating with beta particles. This makes alpha-emitting radionuclides highly potent in targeted radiation therapies [33].

Given the advantages of alpha particles in cancer treatment, one particularly promising application is the ^225^Ac-based PSMA-targeted therapy, which has shown significant potential in treating metastatic castration-resistant prostate cancer. Post-treatment imaging is crucial for monitoring where the radioactive tracer travels within the body and determining the radiation dosimetry; thus, research suggests that carrying out three specific photo peaks, instead of two, during post-treatment imaging protocols significantly enhances imaging quality, ultimately helping to improve treatment monitoring and potentially therapeutic outcomes [35].

LET properties and potential side effects of ^223^Ra, ^177^Lu and ^225^Ac are presented below:

^223^Ra is an alpha-emitting radionuclide with a high LET (>100 keV μm^−1^), densely emitting energy over a short distance (50–100 μm). Common side effects include nausea, fatigue, bone pain, diarrhea, peripheral edema, and bone marrow suppression (leading to low blood cell counts) [36].

^177^Lu emits beta particles with a low LET (about 0.2–0.4 keV μm^−1^), resulting in less intense but more widespread damage to cancerous and surrounding cells. The most common side effects include fatigue, nausea, vomiting, dry mouth and throat, bone marrow suppression, and flushing [37].

^225^Ac is an alpha emitter with a high LET (≈50–200 keV μm^−1^) that delivers substantial energy in a short range (ranging from 40 to 100 μm). However, it potentially causes important cytotoxicity events. Considering this risk, ^225^Ac is frequently coupled with a targeting molecule (such as a monoclonal antibody) in order to enhance its precision. Most common side effects include fatigue, nausea, vomiting, bone pain, decreased appetite, weight loss, and bone marrow suppression, leading to low blood cell counts [33].

## 5. Theranostics with PSMA

PSMA is a type II transmembrane glycoprotein expressed in the prostate cell’s normal epithelium, associated intracellularly with the cytoplasm and extracellularly with the epithelium around prostatic ducts. Neoplastic prostatic tissues transfer PSMA from the apical membrane to the luminal surface of the ducts and cause a 100- to 1000-fold overexpression of PSMA in the prostate neoplasm epithelium in comparison with the native tissue [38,39].

The use of PSMA started with anti-PSMA monoclonal antibodies (Prostascint^®^) over 20 years ago, but its large molecular structure limited its utility. Ample clinical acceptance began in 2012 when smaller molecules were developed, notably the ^68^Ga-PSMA-11, ^18^F-PSMA-1007, and ^18^F-DCFPyL molecules [40,41,42,43]. Since then, several PSMA-based radiotracers have been developed with different chemical structures and radiolabeled with several different radioisotopes, such as ^11^C, ^18^F, ^123^I, ^124^I, ^125^I, ^131^I, ^99m^Tc, ^68^Ga, ^177^Lu, ^44^Sc, ^64^Cu, ^111^In, ^86^Y, ^90^Y, ^225^Ac, ^213^Bi, and ^211^At, although not all of these found their way into clinical practice.

In the Theranostic concept, this overexpression of PSMA is advantageous as it has potential use as a binding target for radioisotopes for both diagnostic PSMA PET/CT images and for therapy.

For diagnostic purposes, PSMA PET/CT images currently available in Brazil are ^18^F-PSMA-1007 and ^68^Ga-PSMA, which use the radioisotopes fluoride-18 and gallium-68, respectively. Although the initial use of ^68^Ga-PSMA has significantly improved the evaluation of patients with prostate cancer, ^18^F-PSMA has practical advantages compared to ^68^Ga-PSMA, such as a longer half-life (110 min versus 68 min, respectively), minimal urinary excretion, thereby facilitating local tumor detection (by reducing urine-related uptake in the prostatic region), and cyclotron production (avoiding the need for an in-house ^68^Ga generator).

PSMA PET/CT allows the selection of suitable patients for treatment with corresponding PSMA-labeled radiopharmaceuticals, such as lutetium-177 (^177^Lu-PSMA) and actinium-225 (^225^Ac-PSMA), as shown in Figure 1.

PSMA expression in the primary tumor has a significant correlation with total PSA and Gleason score [40,42]. Several factors might induce greater PSMA overexpression. Generally, high-grade neoplasms tend to present with higher PSMA expression. As disease progression occurs, it is accompanied by an increase in the number of PSMA-expressing lesions, as well as in their PSMA expression profiles. When advanced stages of disease are reached and as castration-resistance develops, a common phenomenon detected by PSMA PET/CT is a decrease in PSMA expression, which signals that tumor cells are becoming dedifferentiated or going through neuroendocrine differentiation, which might occur in up to 25% of patients [44]. In these settings, ^18^F-FDG PET/CT evaluation becomes more suitable.

A relevant confounding factor regarding PSMA expression that must be mentioned is its poorly understood relation with anti-androgenic therapy administration. While most patients present an overall decrease in PSMA expression, a heterogeneous response is not uncommon, with some lesions increasing PSMA expression, even with adequate serum PSA response. Prospective studies are still underway to better understand this context, but an initial theory suggests that PSMA PET/CT analysis might be able to predict which patients will reach castration resistance earlier when compared with serum PSA monitoring [45].

### 5.1. PSMA Chemical Structure and Ligand Development

The PSMA gene is located on the short arm of chromosome 11 [46] and its cDNA clone encodes a protein of 750 amino acids, of which 19 are from the intracellular portion, 24 from the transmembrane portion, and 707 from the extracellular portion [47].

The chemical structure of PSMA offers valuable information about its catalytic mechanism and substrate specificity. The arrangement of the active site, along with its effects on substrate interaction and catalysis, enhances our understanding of this critical enzyme’s function and guides the development of agents for cancer diagnosis and therapy [48].

One of the enzymatic functions of PSMA is to cleave the substrate N-acetyl-L-aspartyl-L-glutamate (α-NAAG) through a catalytic mechanism [49], whereby α-NAAG binds, leading to the formation of electrostatic interactions between the side chain of glutamate and the arginine region of PSMA. This promotes the binding of the oxygen from the carboxyl group at the C-terminus of α-NAAG to the zinc ions, forming a bridge. This binding is crucial, as it helps to stabilize the interaction between the substrate and the enzyme. The remaining part of the substrate will make specific interactions with the arginine residue (Arg-210). These interactions assist in the positioning of the substrate within the enzyme, facilitating cleavage. Larger substrates may require additional structural rearrangements and may exhibit an alternative binding mode [50].

The mechanism occurs in a similar fashion: the zinc ions present in the active site of PSMA assist in the stability of the chemical reaction. When the substrate binds to the enzyme, a water molecule also binds to the zinc ions. The glutamate residue (Glu-424) present in the enzyme acts as a deprotonating agent for the water molecule, which promotes its activation. Once activated, it attacks the peptide bond between two amino acids present in the substrate that is to be cleaved. This attack creates a temporary structure resulting from the arrangement of atoms during the reaction. This structure is unstable, leading to the cleavage of the peptide bond, resulting in the dissociation of the reaction products, allowing the enzyme to be ready to cleave another substrate molecule [48].

The structure of the active site of PSMA and its implications for substrate binding and the catalytic mechanism provide insights into its enzymatic function and a reasoning for the development of reagents for cancer detection and treatment [48].

Most PSMA ligands that have progressed to clinical use feature a core chemical structure typically consisting of glutamate-urea-glutamate or glutamate-urea-lysine dimers, which are critical for binding to PSMA’s catalytic domain [51]. These ligands often differ in the design of their linkers and chelators while retaining the same fundamental binding motif, allowing for enhanced targeting precision. Additionally, these molecules can be radiolabeled with various positron-emitting isotopes, such as ^68^Ga, ^18^F, and ^64^Cu. Each isotope offers distinct properties, including differences in half-life, positron energy, and synthesis methods [52].

The structural and functional similarities between N-acetyl-α-linked acidic dipeptidase I (NAALADase) and PSMA have facilitated the development of PSMA ligands derived from small-molecule inhibitors of this enzyme [53,54,55]. These structural elements emerged from the gradual development of glutamate-derived hydroxyphosphinyl derivatives, initially designed to inhibit NAALADase in brain tissue [56].

Subsequently, urea-based and phosphonic acid-based PSMA ligands have been investigated, developed, and continuously improved for use in the diagnosis and treatment of prostate cancer through PSMA targeting [57,58,59]. The earliest documented use of PET with small-molecule inhibitors targeting PSMA for imaging prostate cancer in animal models involved a glutamate-urea-cysteine analog [60]. Years later, PET scans using PSMA ligands labeled with ^18^F were performed in humans, demonstrating encouraging outcomes [61,62].

The development of ^68^Ga-PSMA-11 represented a significant clinical advancement in PET imaging using PSMA ligands, as this compound efficiently internalizes into prostate cancer cells due to having a high binding affinity for PSMA [8,63]. Its binding mechanism is similar to other Glu-urea-based PSMA ligands: it attaches to the extracellular domain of the PSMA receptor to be internalized and, since it is a low molecular weight ligand or a “small molecule”, it penetrates tissue easily and effectively diffuses into solid tumors [41,64,65].

### 5.2. PSMA Ligand Targeting Mechanism

Characterized as a type II transmembrane protein, PSMA is also referred as N-acetyl-α-linked acidic dipeptidase I, glutamate carboxypeptidase II (GCPII), or folate hydrolase 1 (FOLH1) [66]. Its chemical structure presents a symmetric dimer, where each polypeptide chain consists of protease, apical, and helical domains. The helical domain plays a crucial role in forming a dimer interface, and only the dimeric form of PSMA is expressed on the surface of prostate cells [67]. Additionally, it exhibits enzymatic activity, functioning as a glutamate carboxypeptidase through a binuclear zinc active site, which catalyzes the cleavage of glutamates from peptides or small molecules [68]. Its enzymatic activity and expression may be reduced due to the restraint or removal of PSMA glycosylation, which may interfere with protein misfolding and dimerization inhibition [67].

Located in the cytoplasm and at the apical region of the prostate epithelium of benign cells, PSMA undergoes a relocation throughout malignancy progression to the prostate duct’s luminal surface, exposing its extracellular domain for ligand binding [69]. The extracellular domain serves as a tumor marker, making it a target for monoclonal antibodies used to deliver specific imaging agents or therapies to cells that overexpress PSMA [70].

PSMA functions as a transport protein due to its involvement in ligand internalization via endocytosis [71], a process facilitated by an internalization signal that enables the protein on the cell surface to be taken into an endosomal compartment. When a ligand binds to PSMA, it is internalized via clathrin-mediated endocytosis [72], a mechanism crucial for vesicular trafficking, facilitating the movement of various molecules from the external environment into the cell [73]. This process enhances the uptake and retention of the molecule within tumor cells and contributes to better imaging quality for diagnosis [55]. Five N-terminal amino acids (MXXXL) in the cytoplasmic tail of PSMA, including methionine and leucine, mediate PSMA internalization. This motif is recognized by adaptor proteins involved in endocytosis, and upon binding to a ligand or therapeutic agent, PSMA is internalized into the endosomal compartment of the cell [72].

Radioligands that target PSMA have hydrophilic properties, leading to rapid and large-volume biodistribution [74], and have no strong binding capacity to blood components. PSMA-negative cells are primarily reached through passive diffusion across cell membranes into the interstitial spaces of organs; however, their hydrophilic nature limits this process, causing most radioligands to remain in the bloodstream.

Cells that express PSMA have surface receptors that capture and internalize PSMA radioligands, leading to their accumulation within the cell’s cytoplasm [11,75]. This mechanism underlines why PSMA is an excellent target for developing small-molecule radiopharmaceuticals, as it can clear rapidly from the bloodstream while producing low background activity [55]. Additionally, research has shown that tumor volume plays a critical role in determining the distribution of these radioligands. Larger tumors tend to have more PSMA receptors, which correlates with higher rates of radioligand uptake compared to smaller tumors [76].

The PSMA active substrate recognition site features two important structural components. One is designed to interact with the glutamate segment found in N-acetylaspartylglutamate (NAAG). This interaction is crucial for the specific binding of ligands that mimic glutamate. The second component consists of a series of basic amino acids, which facilitates the binding to the free carboxylate group of aspartate and allows for the accommodation of larger and more complex structural elements [77]. This dual interaction is essential for the effective recognition and uptake of PSMA-targeting agents [78]. Consequently, designing functionally active molecules that target PSMA is complex, particularly when incorporating bulky chelators for radiometals [55].

### 5.3. Synthetic Strategies of PSMA Analogues

The ^111^In-Caproma antibody, initially used for soft-tissue evaluation, was limited in detecting metastases as it binds to the intracellular region of PSMA [79]. Subsequently, a low-molecular-weight molecule that could attach to the extracellular surface started to be utilized for diagnosis and therapy [80].

Since adding an aromatic group to urea-based PSMA inhibitors like PSMA-11 and PSMA-617 results in a high affinity, these inhibitors have been extensively used in nuclear medicine. Substantial accumulation in the tumor and good in vivo kinetics were seen when squaric acid was added to L-lysine urea-L-glutamate (KuE) for labeling the PMSA-11 and PMSA-617 analogs with ^68^Ga [81].

In the PSMA molecule, the hydrophobic alteration between the glutamate-urea-lysine binding motif and the 1,4,7,10-tetraazacyclododecane-1,4,7,10-tetraacetic acid (DOTA) chelator has produced some interesting results. The PSMA inhibitors conjugated with DOTA based on urea had no reactivity with human serum proteins and exhibited an affinity of over 98% and 95% with ^177^Lu and ^68^Ga, respectively [82]. Non-radioactive analogs can be synthesized through the gentle conjugation used in PET imaging, allowing longer storage times for these compounds. This method involved chelating metals (such as Gd3+La3+, Ce3+Cu2+, Ga3+, In3+, and Y3+) to DOTA and attaching them to the side chains of protected lysines early in the synthesis process [83].

Moreover, creating probes that target cancers with low PSMA expression has been challenging. The hybrid probe JMV 7489, which binds to PSMA and/or neurotensin receptor-1 (NTS1), was synthesized in batch and in a solid phase. The in vitro data showed a good affinity for PSMA; however, this was not the case with NTS1, indicating that the linker and the DOTA macrocycle are essential components of the hybrid molecule [84].

Next, we will discuss the main clinical applications of radiolabeled PSMA.

## 6. Diagnosis with PSMA PET/CT

PSMA PET/CT has been increasingly used in the diagnostic evaluation of patients with prostate cancer due to its high sensitivity in detecting primary lesions as well as metastases.

Applications include primary staging of high and intermediate unfavorable risk prostate tumors; determination of the optimal site for prostate biopsy and radiotherapy (RT) planning; identifying the site(s) of biochemical recurrence; assessment of response to treatment; and selection for PSMA theranostic treatment [85].

### 6.1. Primary Staging

PSMA PET/CT in initial staging is indicated for patients with unfavorable intermediate and high risk, including patients with a Gleason score of 7 (4 + 3) or higher, ISUP 3 or higher, serum PSA greater than 20 ng/mL, or clinical stage T2c-3a [86]. It alters staging in approximately 65% of patients and confers a change in management in approximately 45% [87].

**Figure 7:**
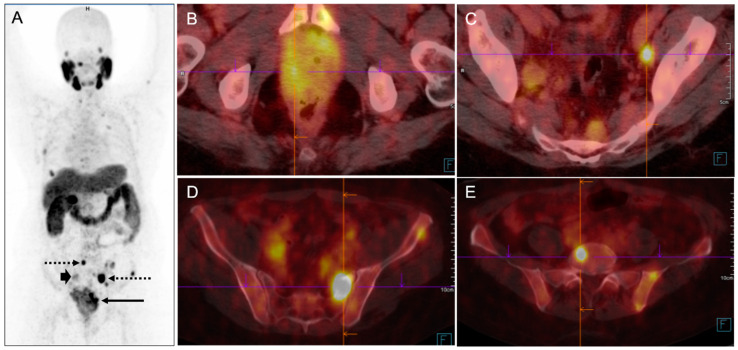
PSMA PET/CT of a patient undergoing primary staging for prostate adenocarcinoma (Gleason 5 + 4 = 9) and PSA= 7.7 ng/mL. (**A**) MIP images show multiple focal regions of hyperexpression of PSMA receptors in the prostate (arrow), lymph node metastases (arrowheads), and bone (dotted arrows). The fused axial images demonstrate (**B**) the primary prostate cancer mass invading adjacent soft tissue and bladder; (**C**) a left internal iliac pelvic lymph node metastasis; (**D**) bone metastasis on the left sacral wing and S4/S5 invading soft tissue; (**E**) a vertebral metastasis. The proposed metabolic staging by molecular imaging (MI) with PSMA was mi T4N2M1c.

Primary staging of unfavorable intermediate and high-risk prostate cancer is essential for accurate lymph node status classification when radical prostatectomy is indicated for adequate surgical planning. It also serves as a prognostic indicator for determining recurrence risk (Figure 7).

Until 2022, according to American guidelines, and until 2020, according to European guidelines, the primary staging of prostate cancer to detect metastatic disease was performed using conventional exams: bone scintigraphy and CT of the abdomen and pelvis. However, these conventional methods usually cannot detect disease when presented with a small tumor burden. For patients with high-risk prostate carcinoma, PSMA PET/CT shows a 27% higher accuracy than CT of the abdomen and pelvis with iodinated contrast associated with bone scintigraphy with SPECT (92% vs. 65%), with higher sensitivity (85% vs. 38%) and specificity (98% vs. 91%). This superiority is also true in the analysis of lymph node metastases and distant metastases. Conventional imaging presented more equivocal findings than PSMA PET/CT (23% vs. 7%) [88].

Local evaluation of the primary tumor in the prostate should be performed with MRI because, due to its high resolution, it allows precise location of the tumor, the detection of extraprostatic invasion, and invasion of seminal vesicles and adjacent organs [89]. Nonetheless, its association with PSMA PET/CT may yield better performance. The association of MRI with PSMA PET/CT, when compared with MRI alone, showed higher sensitivity (97% vs. 83%) and negative predictive value (91% vs. 72%). PSMA PET/CT demonstrated an advantage, mainly in patients with equivocal or negative findings on MRI. Preliminary results also appear promising for the use of PMSA PET/CT as a guidance method for repeat biopsy after a previous negative result [90].

The American and European guidelines currently recommend the use of PSMA PET/CT in the primary staging of intermediate and high-risk prostate cancer [91,92]. The current trend is to increase its use in clinical practice, replacing conventional imaging studies.

### 6.2. Radiotherapy Planning

PSMA PET/CT shows a significant impact on intended definite RT planning for local therapy, ranging between 16.5% and 37% of patients [93], in which the method allowed for better field delineation. It has also been successfully used in radiotherapy planning by identifying metastases distant from the usual field of radiotherapy with curative intent in approximately 48% of patients [88].

The multicenter randomized prospective study, proPSMA, demonstrated that PSMA PET/CT promoted a change in management from curative to palliative intent in 14% of patients. Also, in this study, 7% of the patients had a change in the radiotherapy technique (28). Another study comparing PSMA PET/CT and MRI for radiotherapy planning points to increased consensus of the former with histopathology for intraprostatic gross tumor volume delineation, with higher sensitivity. Furthermore, MRI observer-dependent contours significantly underestimate tumor volume. Thus, PSMA PET/CT has been complementary when seeking to determine tumor volume and to improve radiotherapy results [94].

### 6.3. Detection of Biochemical Recurrence

**Figure 8:**
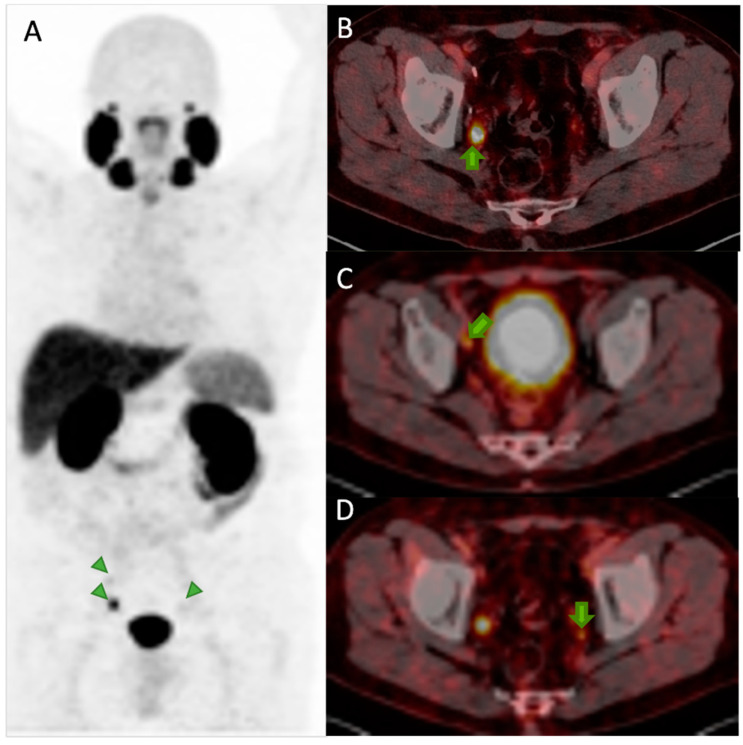
PSMA PET/CT of a patient undergoing restaging due to biochemical recurrence of prostate adenocarcinoma (Gleason 3 + 4; PSA = 0.8 ng/mL). (**A**) The MIP image shows multiple bilateral pelvic lymph node metastases with hyperexpression of PSMA receptors (arrowheads). The fused axial images demonstrate the metastases (green arrows) located in (**B**) a right internal iliac lymph node (0.6 cm; SUV = 11), (**C**) a right external iliac lymph node (0.5 cm; SUV = 3.4) and (**D**) a left internal iliac lymph node (0.3 cm; SUV = 4.2). There were no signs of local recurrence.

In cases of suspected biochemical recurrence, PSMA PET/CT is the most sensitive imaging exam for detecting locoregional and distant metastases, and it is the recommended imaging method [91,92] (Figure 8). Frequently, the appearance of radiopharmaceutical uptake precedes the emergence of anatomical changes; thus, in a great number of patients with low PSA levels, PSMA bridged the gap existing with conventional imaging methods by allowing earlier detection of lesions.

To illustrate, CT sensitivity is approximately 12% for patients with PSA < 1.0 ng/mL and 13% with MRI for patients with PSA ≤ 0.5 ng/mL [95]. In contrast, even for very low PSA values (<0.2 ng/mL), there is considerable PSMA PET/CT positivity, which increases as PSA rises: approximately 45–60% with PSA levels ranging from 0.2 to 0.5 ng/mL; roughly 73% with PSA levels ranging from 0.5 to 1.0 ng/mL; approximately 90% with PSA levels ranging from 1.0 to 2.0 ng/mL; and reaches 95% with PSA > 2.0 ng/dL [96,97]. When analyzed independently of the PSA value, the overall PSMA PET/CT positivity rate in biochemical recurrence approaches 68% [97].

**Figure 9:**
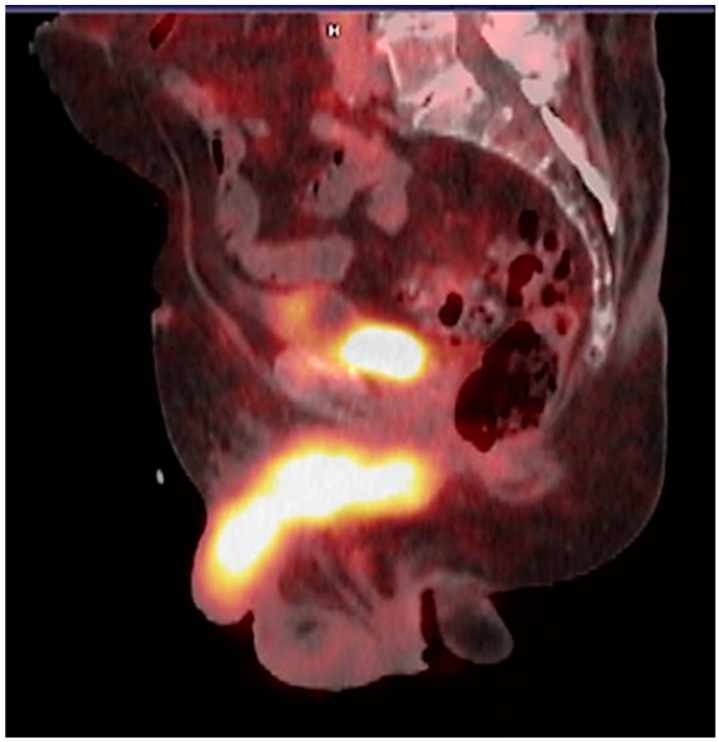
PSMA PET/CT demonstrated an unusual sight of metastasis infiltrating the penis of a patient with prostate adenocarcinoma.

In addition to the identification of common metastatic sites, such as lymph nodes, bone, and lungs, with PSMA PET/CT, it is possible to identify metastases in unusual sites, such as infiltration of the penis, the vas deferens, and the testes (Figure 9), frequently relying solely on the presence of radiopharmaceutical uptake.

The positive predictive value of PSMA PET/CT in biochemical recurrence is close to 100%, which guarantees with great confidence that exams with PSMA-uptake areas indeed represent the sites of viable disease. Impact on patient management occurs in more than 50% of patients; biochemical recurrence-free survival after PSMA PET/CT has been shown to be approximately 60% at a median of 20 months after salvage treatment [98,99,100,101].

### 6.4. Assessment of Response to Treatment

This potential indication of PSMA PET/CT still lacks results from prospective trials, with only limited preliminary data available. One of the main confounders regards the impact of anti-androgenic therapies on PSMA expression, which is not yet fully understood. Similarly, the impact of other treatment strategies also needs additional clarification [102].

Nevertheless, the use of molecular response criteria over morphological changes seems promising, and a baseline study is recommended prior to the introduction of systemic therapies, especially anti-androgenic ones [103].

## 7. Treatment with Radiolabeled PSMA

PSMA is a powerful and specific target for the treatment of prostate cancer due to its high expression in tumoral cells. Currently, the main radioisotopes to which this molecule can be labeled are lutetium-177 (^177^Lu) and actinium-225 (^225^Ac). Both will be discussed in this next section.

### 7.1. ^177^Lu-PSMA

**Figure 10:**
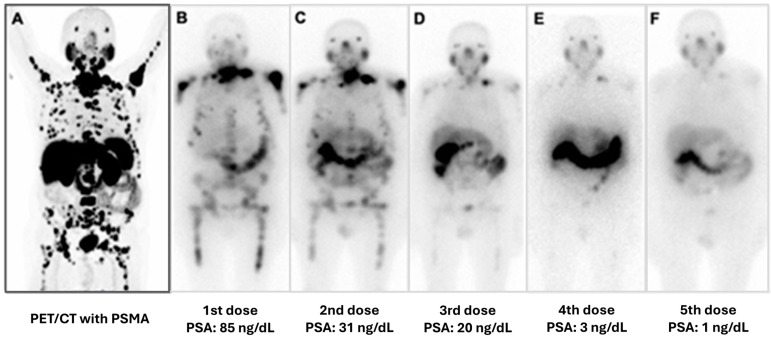
Example of the Theranostic concept (molecular radiolabeling for diagnostic and therapy purposes). In this case, the same molecule (PSMA) was radiolabeled with two different isotopes (gallium-68 and lutetium-177). ^68^Ga-PSMA was used for diagnosis of the extent of metastases from prostate cancer to plan therapy with ^177^Lu-PSMA (**A**) ^68^Ga-PSMA PET/CT image of a patient with extensive, wide-spread bone metastases from advanced prostate cancer with high PSMA uptake. Because of the avidity of the cancer cells to PSMA, the patient was programmed for personalized therapy with ^177^Lu-PSMA. (**B**–**F**) images obtained 4 h after therapy showing the extent of bone metastasis with an initial PSA level of 85 ng/dL. In these subsequent cycles, the images obtained show excellent response to treatment. The patient presented with a progressive reduction of PSA levels, improvement of symptoms and performance status, and significant reduction of bone metastasis.

^177^Lu-PSMA provides a therapeutic effect due to the emission of high-energy beta particles, which cause damage to PSMA-expressing tumor cells via single-strand DNA breaks. The treatment is performed intravenously in doses of 7.4 GBq (200 mCi), once every six weeks, for 4 to 6 cycles. Its radioprotection profile allows for administration in an outpatient setting (Figure 10).

The scenario in which the treatment is already well established (evidence category 1A) is for metastatic castration-resistant patients (mCRPC). The pivotal Vision trial [104], a randomized phase III study published in the New England Journal of Medicine in 2021, compared ^177^Lu-PSMA to standard therapy in metastatic castration-resistant prostate cancer patients who had exhausted all treatment options. Patients in the ^177^Lu-PSMA arm showed favorable and significant outcomes in terms of overall survival, disease-free progression, and radiological progression compared to standard therapy.

Selection criteria for ^177^Lu-PSMA therapy is crucial for better outcomes, and PSMA PET/CT plays an important role, as it allows the detection of lesions with high PSMA expression that can serve as potential targets. However, selection criteria have not yet been standardized, and discrepancies exist among different clinical trials. The Vision study used broader criteria, while other studies were more restrictive. For example, the TheraP study in Australia employed criteria based on the presence of PSMA-positive lesions with SUV > 20 and also included FDG PET/CT imaging, excluding patients with discrepancies between the two exams (i.e., FDG-positive lesions and PSMA-negative lesions) [105].

Although adding FDG PET/CT presents additional logistical and financial challenges, this option can be beneficial in identifying PSMA-negative metastases, which are associated with worse prognosis [106]. According to the SNMMI/EANM guidelines, FDG PET/CT is considered beneficial but not mandatory [86].

Regarding PSMA PET/CT, there is no specific uptake limit for patient selection. However, the exclusion of patients with lesions >1 cm and who show low uptake compared to the parotid gland uptake is suggested [107].

The guideline also recommends PSMA PET/CT as the ideal imaging modality for assessing therapy response, with the CT component preferably following a dedicated diagnostic protocol for evaluating potential PSMA-negative lesions. Complementary methods, such as FDG PET/CT and/or bone scintigraphy, should be considered on a case-by-case basis for response assessment. The standardization of response criteria based on imaging remains an ongoing challenge. Nevertheless, there is great anticipation that as theranostic medicine becomes more widespread and ongoing clinical trials are consolidated, the necessary tools for proper patient monitoring through imaging will be validated in the near future.

^177^Lu-PSMA clinical trials have increased in number. Small prospective studies determined adequate treatment safety for castration-sensitive patients (CSPC) [108]. Larger prospective trials will determine the therapy potential earlier in the course of disease progression. These trials include: LuTectomy (^177^Lu-PSMA before prostatectomy after initial staging in patients with high-risk localized disease—NCT04430192), PSMAddition (^177^Lu-PSMA combined with anti-androgen therapy vs. isolated anti-androgen therapy for CSPC—^177^Lu-PSMA—NCT04720157), UpFront PSMA (^177^Lu-PSMA followed by chemotherapy vs. isolated chemotherapy for mCSPC—NCT04343885), and PSMAfore (^177^Lu-PSMA vs. alteration of anti-androgen therapy for pre-chemotherapy mCRPC—NCT04689828), among several others.

### 7.2. ^225^Ac-PSMA

^225^Ac-PSMA is an alpha radiation emitter. Compared to beta particles, alpha particles contain higher energy and travel shorter distances from the nucleus, which provides greater efficiency in destroying cells even at lower concentrations (LET phenomenon), and at the same time leading to lower toxicity as it radiates less surrounding healthy tissue. Alpha particles cause irreversible damage to tumor cells by breaking both strands of DNA, causing cell cycle arrest, apoptosis, and necrosis. Its main disadvantage is that worldwide potential for production is low, meaning that its use in clinical practice is still unfeasible. Currently, the use of ^225^Ac-PSMA is restricted mainly to research in a few centers around the globe.

Data from the meta-analysis show that approximately 37% of patients treated with ^177^Lu-PSMA will be refractory and will present biochemical progression [109]. The ongoing prospective trials regarding ^225^Ac-PSMA aim to assess if its superior damage potential translates into better outcomes for these patients. Preliminary results are promising, demonstrating adequate response with fewer side effects. Nevertheless, more robust data regarding its strengths and limitations are needed, as well as a global improvement in the radioisotope production chain [110,111].

## 8. Conclusions

Nuclear medicine plays an increasingly significant role in the diagnostic and therapeutic approach to urological malignancies.

The enormous advances in SPECT/CT and especially PET/CT images now allow an assessment of these tumors in the staging, recurrence, and response to treatment settings.

Molecular imaging identifies alterations not identified by anatomical imaging and, for this reason, PET/CT images are becoming increasingly indispensable in specific clinical situations and with precise indications according to the type of urological neoplasia.

Theranostic Nuclear Medicine is rapidly evolving in prostate cancer and is a well-established and routine treatment option.

Theranostic Nuclear Medicine is a personalized therapy. The concept of using the same molecule for diagnosis and therapy opened the door to guided and effective treatment, especially for metastatic castration-resistant patients. This increased survival and preserved quality of life with low side effect rates. There is great hope that the field will also encompass earlier stages of the disease in the future.
